# Effects of haze pollution and institutional environment on demand for commercial health insurance

**DOI:** 10.3389/fpsyg.2022.1002470

**Published:** 2022-11-25

**Authors:** Pan Jia, Jingshi Yan

**Affiliations:** School of Finance, Southwestern University of Finance and Economics, Chengdu, China

**Keywords:** haze pollution, risk awareness level, institutional environment, commercial health insurance, China household finance survey

## Abstract

What countermeasures should the public take as they become aware of the dangers of haze pollution? Insurance has the function of risk diversification, and little existing literature has focused on the relationship between haze pollution and commercial health insurance. This paper analyzes the impact of haze pollution on residents’ demand for commercial health insurance and the heterogeneous impact of institutional environment using the 2017 China Household Finance Survey cross-sectional data (CHFS). The study finds that haze pollution raises residents’ demand for commercial health insurance as their health risk perception level rises. The legal environment, market environment, and traditional culture affect the relationship between haze pollution and the demand for commercial health insurance. Further analysis reveals that the relationship between haze pollution and residents’ demand for commercial health insurance can show significant regional heterogeneity, with a significant positive correlation in the eastern region and a significant negative correlation in the central and western regions. In addition, the preventive behaviors adopted by residents in the face of haze pollution can vary significantly depending on individual risk preferences. The findings of this paper are important for the public to take measures to cope with the haze pollution hazards. At the same time, insurance companies should improve their services to meet the needs of the public regarding haze pollution, which will contribute to the healthy development of the insurance industry.

## Introduction

The impact of air pollution on residents’ physical and mental health cannot be underestimated, and can directly and indirectly cause short-term and even long-term damage to human functions, which in turn increases the health costs of residents and shortens life expectancy *per capita* (Chen et al., 2013). Air pollution causes mostly physiological diseases, and severe air pollution increases the incidence of lung cancer and other respiratory diseases (Li et al., 2018) and air pollution ranks fourth among the causes of death in China (Wang et al., 2012), and respiratory diseases are also the fourth cause of death from diseases. Song et al. (2019) found haze pollution affects public well-being through its effect on public mental health. Since 2010, when the first entry of “PM2.5” was established in Baidu Encyclopedia, the concern and attention to air pollution has gradually increased in China. This shows that the public’s awareness of eco-environmental protection and their own health concerns are gradually increasing. As a risk management tool, insurance is a behavioral choice for the insured to deal with uncertainty. Combined with behavioral finance theory, we know that the decision to purchase insurance is the result of a combination of risk perception, risk management, insurance cognition, and insurance burden, and that this behavior process begins with risk perception. Once the public perceives that their health may suffer from the haze, they may share the risk through health insurance. Therefore, an in-depth analysis of the influencing factors of commercial health insurance is important for the public to cope with risks and promote the development of China’s insurance industry.

Currently, most scholars have only discussed the relationship between individual characteristics and risk perceptions. In this paper, we try to analyze the link between the two in a more macro-level institutional context, such as gender, age, income, health status, and education level, will affect their risk cognition level. Most of these characteristics, except gender and age, are subject to the institutional environment. Because the institutional environment surrounds civil society, every step of development must be directly or indirectly affected by the institutional environment. Various formal or informal systems ultimately shape the form and characteristics of civil society (Song et al., 2019). In other words, most of the individual characteristics of the public are determined by the institutional environment. Moreover, a good institutional environment can also reduce the information asymmetry between subjects by establishing effective legal mechanisms, providing clean and efficient public governance, giving full play to the role of the market in resource allocation (Deng et al., 2014), and improve the public’s awareness of health risks. Therefore, the role of the institutional environment should be fully considered in analyzing the public consumption behavior of insurance products.

Compared with the existing literature, this paper is innovative in the following aspects: first, while the existing literature focuses more on the immediate defensive behavior of the public (Xu et al., 2017), this paper innovatively explores the preventive behavior of the public from the perspective of commercial insurance. Second, although the existing literature found the relationship between haze pollution and commercial health insurance (Wu et al., 2019), it did not explain and justify the mechanism of action. This paper innovatively uses the Baidu index of air purifiers to confirm that the level of risk perception is an important intermediate path for haze pollution to increase public demand for commercial health insurance. Third, this paper extends the analysis at the institutional environment level, and gives micro-level empirical evidence on the risk-averse function played by insurance products in haze pollution events. Compared with studies in other countries, the impact of the institutional environment on commercial insurance has Chinese characteristics, and the findings of this paper are crucial for the development of the Chinese insurance industry.

## Literature and hypotheses

Commercial health insurance has developed rapidly in recent years, but its role in the medical security system is still insignificant because of its low base and low penetration rate. The reasons for this phenomenon can be roughly divided into economic and demographic factors. Hammond et al. (1967) found that increased family income and net assets increase the probability of family commercial insurance purchases. Showers and Shotick (1994) found that insurance demand was positively affected by the age of the head of the household and the family size. According to the theory of planned behavior, the influence process of economic and demographic characteristics on residents’ purchase of commercial insurance can be summarized as the logical relationship of “risk cognition response behavior” (Brewer et al., 2004; Dominicis et al., 2015; Ferrer and Klein, 2015; Wu et al., 2019). Risk cognition is the driving factor for residents to adopt risk response behavior, and risk coping behavior is the concrete manifestation of risk cognition.

In haze pollution events, how the public responds depends on their awareness of the hazards of haze. Xie et al. (2014) analyzed the impact of haze pollution on residents’ health using the air data of Beijing from January 10 to 15, 2013 and found that a high concentration of haze pollution can cause respiratory diseases such as acute bronchitis and asthma. Moreover, haze pollution will even increase the incidence of lung cancer (Iii and Arden, 2002; Chang et al., 2018). Sun et al. (2022) found consistent associations between haze exposure and acute mental illness, cardiovascular and neurological morbidity and mortality in their study of cross-border flows of haze pollution. It is precise because the public can effectively feel the existence of haze risk, which improves the public’s awareness of health risks and urges residents to take coping behavior (Sun et al., 2022). In the short term, residents can alleviate this harm by reducing outdoor activities, purifying indoor air, and increasing the frequency of mask use (Yuming et al., 2015). However, haze pollution is challenging to eliminate quickly, and residents will be exposed to haze pollution for a long time. Combined with the risk avoidance function of insurance, whether the public will convert health risk cognition into insurance product demand is very important for developing commercial health insurance. Some literature (Hines et al., 1987) has found a positive correlation between air quality and health insurance demand, but few scholars in China pay attention to such problems. Therefore, the first research hypothesis of this paper:

*Hypothesis 1 (H1)*: Haze pollution will promote public demand for commercial health insurance.

The perception of air quality drives the public’s response to environmental pollution, and the formation of risk perception depends on people’s access to environmental information (Bresnahan et al., 1997; Bamberg and MöSer, 2007; Wu and Li, 2017). An institutional environment surrounds civil society. Various formal (laws, regulations, government administrative policies, etc.) and informal systems (culture, customs, etc.) will affect the public’s access to information (Song et al., 2019). Lo (2013) found that the social and cultural environment affects personal perception of risk. Naturally, residents’ access to information on haze pollution risk cannot eliminate the impact on the institutional environment. An excellent institutional environment helps the news media transmit information timely, comprehensively, and effectively (Yang and Chen, 2010), reduce the information asymmetry between subjects, meet the public’s information needs, and improve the public’s awareness of health risks. It can be said that an excellent institutional environment is a prerequisite for the healthy development of the insurance industry. Through empirical analysis, Liu et al. (2010) confirmed that the total provision of information could significantly improve micro health insurance demand. Xu et al. (2017) found through the questionnaire that the higher the public’s perception of haze risk and the more thoroughly they understand haze information, they will take more active protection and response measures. Based on the relationship between institutional environment, haze pollution, and commercial health insurance demand, the second assumption of this paper is:

*Hypothesis 2 (H2):* The institutional environment will affect the relationship between haze pollution and the demand for commercial health insurance.

The development of China’s insurance industry shows noticeable regional differences. From the insurance density and insurance depth perspective, the development speed of the eastern region is significantly faster than that of the central and western regions. The existing literature (Zhang et al., 2005; Fan and Wang, 2015; Suo et al., 2015) shows an apparent regional imbalance in the insurance market development. The reason lies in the difference in regional economic development levels. In addition, the difference in social security level is also an important factor causing this phenomenon. However, few scholars pay attention to the role of the institutional environment. Xiao (2007) found that the difference in the development level of the insurance market among regions in China is relatively small due to the difference in economic development level among regions. Furthermore, the factors leading to the difference in the development level of the insurance market among regions are the economic environment, social and cultural environment, legal environment, insurance market environment, and other factors. Similarly, Yan et al. (2021) and Xu et al. (2021) found that low-carbon city construction was overall effective in reducing air pollution in China, but there was significant geographical heterogeneity in this effect. Considering the apparent differences in social systems in different regions, especially between the eastern and central, and western regions, combined with the analysis of hypothesis 2, the third hypothesis of this paper is derived:

*Hypothesis 3 (H3):* The relationship between haze pollution and the demand for commercial health insurance will show obvious regional heterogeneity.

## Research design

### Specific performance of “risk cognition response behavior” in haze pollution events

Risk cognition is the internal driving factor of coping behavior, and coping behavior is the specific manifestation of risk cognition. Since it is hard to directly quantify the public’s awareness of the risk of haze pollution, we use the Baidu Index of “air purifiers” to measure it indirectly. Generally speaking, the public will pay more attention to the corresponding countermeasures when they fully understand the hazards of haze pollution promptly. Therefore, the larger the Baidu Index of “air purifiers,” the higher the public’s awareness of the risk of smog.^1^

**Figure 1:**
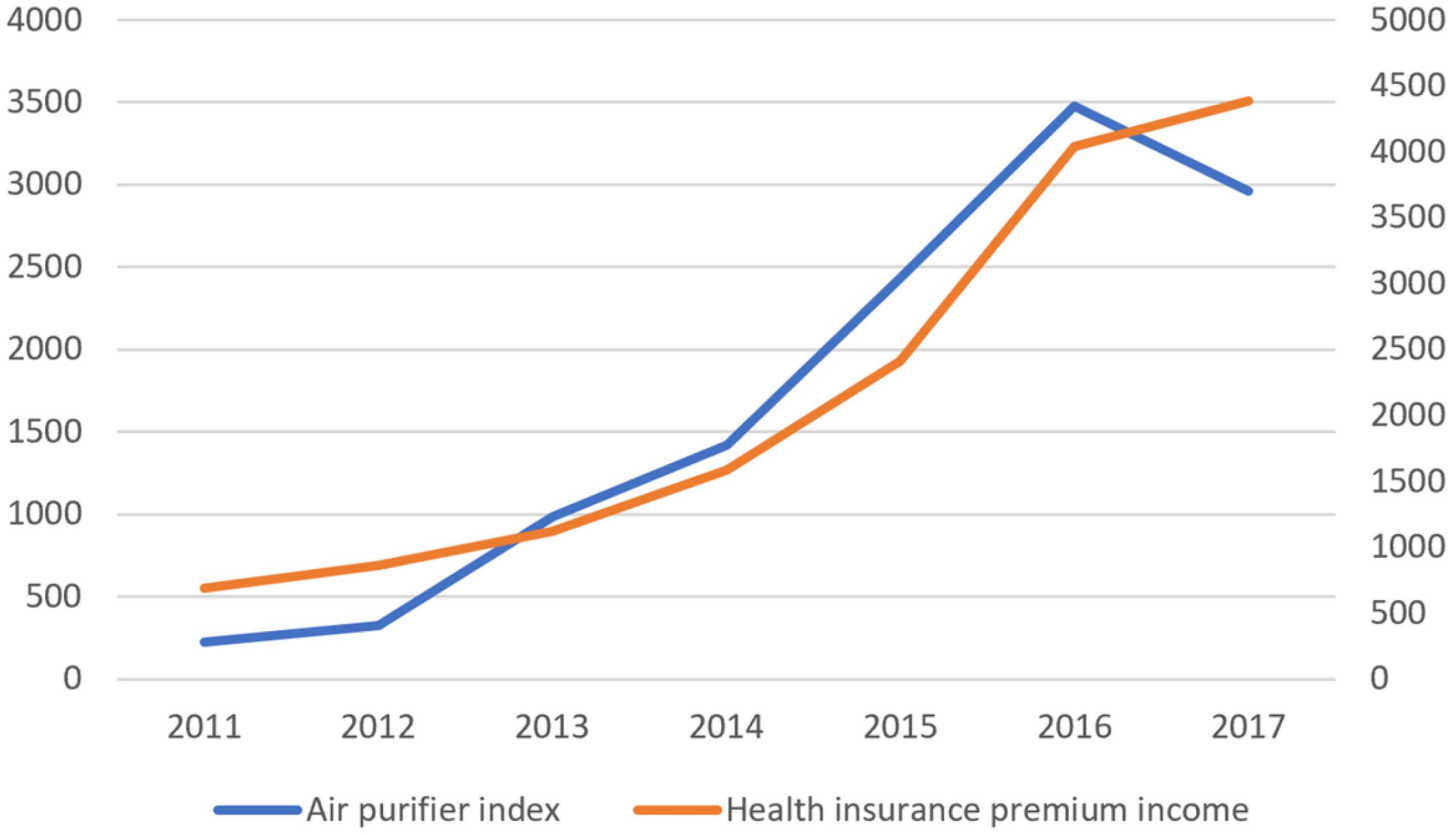
Relationship between health risk cognition and coping behavior. The left vertical axis of the figure is the premium income of commercial health insurance, and the right vertical axis is the moving daily average of the Baidu Index of air purifiers. The premium income data comes from the premium income data of insurance companies on the National Bureau of statistics website.

As shown in Figure 1, from 2011 to 2016, the Baidu Index of “air purifiers” and the premium income of commercial health insurance have shown a geometric growth since 2011, and the growth trend is almost the same. The coupling of the two development trends can preliminarily judge that improving the public’s awareness of health risks has effectively transformed into insurance demand, promoting the rapid development of commercial health insurance. From 2016 to 2017, the Baidu Index of “air purifiers” showed an apparent downward trend related to the achievements of the Chinese government in environmental governance in recent years. The haze pollution improvement has reduced the Baidu Index of “air purifiers.” At the same time, the growth rate of premium income has also slowed down. The recoupling of the two development trends further verifies the importance of health risk awareness level to the development of commercial health insurance, which supports explaining haze pollution and promotes the demand for commercial health insurance.

### Model

To discuss the influence of haze pollution on family commercial health insurance purchasing behavior, In this paper, we construct the following Probit model by drawing on Yan et al. (2021):


$$\mathrm{Pr}\left(\mathrm{Insurance}=1\right)=\emptyset \left(\alpha +\beta_{1}PM2.5+\beta_{2}X+\varepsilon \right)$$

X is the control variable, including family, demographic, and regional economic characteristics. It should be noted that the existing literature found that when individuals perceive that environmental pollution may bring harm to their health, people will pay more attention to environmental problems and are more willing to participate in environmental protection (Xu et al., 2021). However, after the public purchases commercial health insurance, it can produce obvious moral hazards and reduce people’s environmental protection behavior. In other words, there may be an endogenous problem of reverse causality between haze pollution and residents’ purchase of commercial health insurance. Therefore, this paper will use instrumental variables to deal with this problem. At the same time it is considering the effect of household budget constraints, if a household purchases life insurance, it is likely to reduce the probability of purchasing health insurance. This paper will exclude the effect of the household budget in the robustness test section.

In terms of tool variable selection, the existing literature (Yin et al., 2015; Nardi et al., 2021) shows that the formation of haze weather has both socio-economic and natural factors. However, socio-economic factors in this paper do not meet the conditions for using instrumental variables. Therefore, this paper uses the regional average annual temperature as the instrumental variable of haze pollution. Because the regional temperature is related to the haze weather, there is no apparent connection with the residents’ purchase of commercial health insurance. In the robustness test part, this paper also uses the regional average annual temperature and wind speed as the instrumental variables of PM2.5 to test the conclusion again.

### Sample and data

The dependent, independent, and control variables are from the fourth round of the China Household Finance Survey project conducted nationwide by the Southwestern University of Finance and Economics in 2017. The samples are distributed in 29 provinces (autonomous regions and municipalities directly under the central government), 355 counties (districts and county-level cities), and 1,428 Village (neighborhood) committees across the country. A total of 40,011 households’ microdata are obtained, representing the national and inter-provincial levels. China Household Finance Survey collects household assets and liabilities, income and expenditure, insurance and security, and demographic characteristics. Other provincial-level variable data are collected manually through the China Research Data Service platform (CNRDS), the Statistical Yearbook published by each province, and the website of the CBRC.

### Variables

#### Dependent variables

Commercial health insurance demand measurement (Insurance): This paper uses the answers to the third part of the CHFS (2017) questionnaire on the purchase of commercial insurance by family members as the basis (there are four answers to this option: commercial life insurance, commercial health insurance, other commercial insurance and none), and takes the family as the research object. If only one family member has purchased commercial health insurance, the value of insurance is 1; If none of the family members have purchased commercial health insurance, the value is 0.

#### Independent variables

1. Haze pollution measurement (PM2.5). The commonly used index to measure haze pollution is PM2.5 (particles with a diameter of fewer than 2.5 microns are called PM2.5, also known as inhalable lung particles). This paper uses the annual average PM2.5 data of each province provided by the China Research Data Service platform (CNRDS) as the measurement index to measure the haze pollution level. Considering that health risk cognition is a gradual accumulation process, this paper uses the mean value of PM2.5 in each province in the past 3 years as the primary independent variable. The higher the value, the worse the haze pollution; otherwise, the better the haze pollution. To ensure that the analysis results are not affected by the selection of years, this paper will use the PM2.5 mean value of the past 5 years in the robustness test part to verify the conclusion of this paper again, and the other similar variables will be treated the same. This paper, PM2.5 is divided into high and low groups according to the median. In areas more heightened than the median, the value of PM2.5 is 1; For areas below the median, the value of PM2.5 is 0.
2. Institutional environment measurement. This paper measures the institutional environment from two levels: the formal and informal systems. In terms of the formal system, this paper uses the legalization level (Law) and financial industry marketization index (market) of various regions in China compiled by Wang and Fan (2018) to measure the regional formal system environment. The higher the score, the better the formal system environment. On the contrary, the worse the institutional background, The informal system is measured by the number of Confucius and Buddhist temples in each province. The greater the value, the stronger the traditional cultural atmosphere in the region. The samples are also divided into high and low groups for the institutional environment variables by using the median. The high-value group is 1, and the low-value group is 0.

#### Control variables

Referring to the existing literature (Zhang et al., 2010, 2017; Wang and Fan, 2018), this paper also controls other factors affecting the family’s business health needs, including the family’s total income, net assets, family size, the family’s urban and rural region and the economic environment of the family’s region (GDP).As well as the age and age square of the head of household, education, gender, marital status, and health.^2^^,^^3^^,^^4^

## Results

### Descriptive statistics

The mean value of Insurance is 0.05, which means that only 5 out of 100 households have purchased commercial life insurance, commercial health insurance, and other commercial insurance, this value indicates that Chinese residents are not sufficiently aware of purchasing insurance on the one hand, and the insurance market in China has an ample development space on the other hand; the mean value of PM2.5 is The mean value of PM2.5 is 0.52, which indicates that air pollution exists in most of China, and the standard deviation is 0.5, which indicates that the haze pollution varies significantly between cities (Table 1).

**Table 1:** Descriptive statistics.

Variable Name	Sample size	Mean	Standard deviation	Min.	Median	Max.
Insurance	40,011	0.05	0.22	0	0	1
PM2.5	40,011	0.52	0.5	0	1	1
Temp	40,011	0.51	0.5	0	1	1
Income	39,184	10.62	1.53	0	10.92	15.42
Asset	38,471	12.6	1.94	0	12.85	17.22
Health	40,011	3.17	1.55	1	3	15
Age	40,000	55.2	14.25	3	55	117
Gender	40,010	1.21	0.4	0	0	1
Marry	39,966	2.4	1.24	0	0	1
Education	39,958	9.27	4.16	0	9	22
GDP	40,011	10.23	0.77	7.87	10.33	11.4
Rural	40,011	0.32	0.47	0	0	1
Train	40,011	0.1	0.29	0	0	1

### An empirical analysis of the impact of haze pollution on demand for commercial health insurance

Based on the Probit model, this paper uses instrumental variables to solve the possible endogenous problems in the model. Therefore, the Probit + IV model is selected for empirical analysis. Table 2 presents the analysis results of the impact of haze pollution on demand for commercial health insurance. Columns (1) and (2) respectively show the empirical analysis results without introducing control variables and regional effects, and column (3) shows the results after considering both control variables and regional impact. The coefficient of PM2.5 in columns (1) and (2) is significantly positive at the level of 5% and 1%, respectively, and the coefficient of PM2.5 in column (3) is significantly positive at the level of 1%, which is consistent with the expectation of this paper, that is, haze pollution increases the possibility of residents to buy commercial health insurance, and this conclusion will not change due to control variables and regional effects. In addition, the Wald test results reject the hypothesis that there is no endogenous relationship between haze pollution (PM2.5) and the demand for commercial health insurance.

**Table 2:** Impact of haze pollution on demand for commercial health insurance.

Variable Name	(1)	(2)	(3)
	Ivprobit	Ivprobit	Ivprobit
PM2.5	0.177**	0.397***	0.505***
	(2.381)	(5.491)	(5.608)
Income		0.116***	0.119***
		(9.684)	(9.893)
Asset		0.093***	0.098***
		(10.152)	(10.428)
Health		0.057***	0.055***
		(6.586)	(6.339)
Age		0.019***	0.018***
		(3.180)	(3.040)
Age2		−0.000***	−0.000***
		(−6.333)	(−6.202)
Gender		0.043	0.040
		(1.442)	(1.321)
Marry		0.044***	0.045***
		(3.541)	(3.593)
Education		0.026***	0.026***
		(6.939)	(6.958)
GDP		0.009	0.049***
		(0.479)	(2.617)
Rural		−0.114***	−0.114***
		(−3.442)	(−3.409)
Area	Yes	No	Yes
_cons	−1.717***	−4.950***	−5.560***
	(−42.858)	(−18.480)	(−19.464)
*N*	40,011	37,661	37,661
Pseudo *R*^2^	0.079	0.176	0.321
Wald test	3.38	30.50	38.40
Value of *p*	0.066	0.000	0.000

The values in brackets are *Z* values; *, **, *** It is significant at the level of 10%, 5%, and 1%, respectively.

In the control variables, the coefficients of family income and family net assets are significantly positive at the level of 1%, which means that the increase of family income and net assets will improve the possibility of families buying commercial health insurance. The coefficient of education level is significantly positive at the level of 1%, indicating that high-level education helps to improve the current situation of insufficient demand for commercial health insurance. There is an inverted “U” relationship between the age of the household head and the possibility of commercial insurance participation. The gender of the head of household has no significant impact on the purchase of commercial health insurance. Residents with higher health are more likely to buy commercial insurance. This may be because there are specific access conditions in the health insurance market, that is, residents who meet the health indicators are eligible to buy health insurance; The possibility of purchasing commercial insurance in rural areas is significantly lower than that in urban areas; The development of the regional economy will promote the development of commercial health insurance. Overall, the impact of control variables on demand for commercial health insurance is consistent with existing literature’s conclusions (Hammond et al., 1967; Sun and Huang, 2010; Wang and Fan, 2018).

### The impact of haze pollution on demand for commercial health insurance: An analysis based on the institutional environment

#### Analysis based on the formal institutional environment

The institutional environment includes both formal and informal institutional environments. Firstly, this paper analyzes the impact of the formal institutional environment on the relationship between haze pollution and commercial health insurance demand. The coefficient of PM2.5 in columns (1) and (3) of Table 3 is significantly negative at the 1% level, and the coefficient of PM2.5 in columns (2) and (4) is significantly positive at the 1% level. This shows that the difference between the legal environment and the market environment makes the impact of haze pollution on demand for commercial health insurance show apparent heterogeneity. In an excellent formal institutional environment, haze pollution will increase the possibility of residents buying commercial health insurance; on the contrary, it will reduce this possibility.

**Table 3:** The impact of haze pollution on demand for commercial health insurance: an analysis based on the formal system.

Variable name	Legal environment		Industry marketization	
	(1)	(2)	(3)	(4)
	Low-value group	High-value group	Low-value group	High-value group
PM2.5	−3.916***	0.154***	−0.809***	0.154***
	(−3.696)	(3.062)	(−3.510)	(3.271)
Income	0.097***	0.119***	0.124***	0.112***
	(4.771)	(7.239)	(6.931)	(6.933)
Asset	0.170***	0.107***	0.126***	0.105***
	(7.106)	(8.899)	(8.398)	(8.385)
Health	0.070***	0.054***	0.038***	0.054***
	(4.224)	(4.493)	(2.978)	(4.587)
Age	0.022**	0.024***	0.015*	0.027***
	(1.992)	(3.168)	(1.764)	(3.235)
Age2	−0.000***	−0.000***	−0.000***	−0.000***
	(−3.620)	(−5.452)	(−3.526)	(−5.559)
Gender	0.039	0.060	0.055	0.055
	(0.682)	(1.565)	(1.213)	(1.354)
Marry	0.009	0.057***	0.036**	0.043**
	(0.397)	(3.551)	(2.003)	(2.441)
Education	0.014*	0.031***	0.024***	0.028***
	(1.925)	(6.320)	(4.132)	(5.386)
GDP	1.139***	0.189***	0.216***	0.217***
	(3.776)	(5.582)	(3.374)	(6.540)
Rural	−0.186***	−0.100**	−0.151***	−0.096**
	(−3.413)	(−2.078)	(−3.152)	(−2.068)
Area	Yes	Yes	Yes	Yes
_cons	−13.894***	−7.245***	−6.622***	−7.395***
	(−5.651)	(−15.475)	(−10.645)	(−15.866)
*N*	16,499	21,162	17,400	20,261
Pseudo *R*^2^	0.458	0.712	0.469	0.727
Wald test	18.00	8.28	11.03	10.64
Value of *p*	0.000	0.004	0.001	0.001

The values in brackets are *Z* values; *, **, *** It is significant at the level of 10%, 5%, and 1%, respectively.

The public’s ideology is subject to the institutional environment. Under different institutional environments, the publics’ risk cognition level will naturally differ, resulting in this heterogeneity. Improving the legal and market environments in an environment with a poor formal system will help alleviate the information asymmetry between subjects. The public can obtain information timely and comprehensively, enhance risk cognition, and promote commercial health insurance development. With the gradual transformation of government functions and the continuous innovation of social management methods, market-oriented means to meet the needs of social management and public services have become an inevitable choice. To fully play the critical role of commercial insurance in the social security system, we must create a good institutional environment.

Although an excellent social environment system can improve the public’s risk perception level and promote the healthy development of the insurance industry. However, if consumers’ rights and interests are not fully guaranteed, and consumers distrust insurance products, even if the public’s risk perception level is improved, it is difficult to convert this perception into insurance demand effectively. In other words, whether consumers’ rights and interests can be fully protected is a prerequisite for effectively converting risk perception into insurance demand. Therefore, we further examine the protection of consumer rights and interests under the formal institutional environment. In this paper, the number of administrative fines published on the China Banking and Insurance Regulatory Commission (BRC) website in each region is used to measure the level of consumer protection in the region. Generally speaking, the more administrative fines, the higher level of consumer protection in the region, and the healthier the industry development.

Table 4 analyzes the relationship between the formal system and the industry supervision level. It can be seen that the average value of industry fines in the high-value group of the legal environment is 138.825, and the average value in the low-value group is 69.821, and the difference between the two is significant at the level of 1%; In the market environment, the average value of industrial fines in the high-value group is 141.805 and 69.873 in the low-value group. The difference between the two is also significant at 1%, which shows that the better the formal institutional environment, the higher the level of industrial supervision. Only when the rights and interests of consumers can be fully protected can the public’s awareness of risk be effectively replaced by insurance demand.

**Table 4:** The relationship between formal institutional environment and industry supervision level.

Variable Name	Low-value group sample size	Low-value group mean	High-value group sample size	High-value group mean	Inter group mean difference *t*-test
legal environment	17,900	69.821	22,111	138.825	−69.004***
Market environment	18,829	69.873	21,182	141.805	−71.932***

The values in brackets are *Z* values; *, **, *** It is significant at the level of 10%, 5%, and 1%, respectively.

#### Analysis based on the informal institutional environment

Table 5 shows the impact of cultural differences in the informal system on the relationship between haze pollution and the demand for commercial health insurance. The coefficient of PM2.5 in columns (1) and (3) is significantly positive at the level of 1%. In contrast, the coefficient of PM2.5 in columns (2) and (4) is not significant, which indicates that the impact of haze pollution on household commercial insurance purchase behavior will change due to differences in traditional culture, which is consistent with the second hypothesis of this paper.

**Table 5:** The impact of haze pollution on demand for commercial health insurance: an analysis based on the infomal system.

Variable name	Confucian culture (confucian temple)		Buddhism (temple)	
	(1)	(2)	(3)	(4)
	Low-value group	High-value group	Low-value group	High-value group
PM2.5	0.996***	0.123	11.393***	0.205
	(5.076)	(1.630)	(2.978)	(1.209)
Income	0.154***	0.108***	0.174***	0.108***
	(7.855)	(6.942)	(5.382)	(6.546)
Asset	0.078***	0.117***	−0.085	0.097***
	(5.417)	(9.104)	(−1.203)	(7.151)
Health	0.031**	0.055***	0.040*	0.065***
	(2.217)	(4.926)	(1.740)	(5.177)
Age	0.021**	0.022***	0.040**	0.021***
	(2.365)	(2.648)	(2.414)	(2.691)
Age2	−0.000***	−0.000***	−0.001***	−0.000***
	(−4.581)	(−4.792)	(−3.917)	(−4.813)
Gender	−0.078*	0.125***	−0.125	0.053
	(−1.664)	(3.023)	(−1.224)	(1.304)
Marry	0.043**	0.045**	0.026	0.059***
	(2.358)	(2.548)	(0.857)	(3.505)
Education	0.015**	0.027***	0.003	0.036***
	(2.318)	(5.260)	(0.261)	(7.108)
GDP	−0.091***	0.034	−3.014***	0.066*
	(−2.824)	(0.880)	(−2.875)	(1.777)
Rural	−0.148***	−0.111**	−0.443***	−0.114**
	(−2.730)	(−2.574)	(−3.407)	(−2.324)
Area	Yes	Yes	Yes	Yes
_cons	−4.395***	−5.431***	17.987**	−5.741***
	(−10.253)	(−10.880)	(2.230)	(−11.508)
*N*	18,529	19,132	17,982	19,234
Pseudo *R*^2^	0.278	0.503	0.591	0.274
Wald test	26.85	5.37	26.17	3.68
Value of *p*	0.000	0.021	0.000	0.055

The values in brackets are *Z* values; *, **, *** It is significant at the level of 10%, 5%, and 1%, respectively.

Traditional culture, especially the “filial piety” culture in Confucian culture, emphasizes the importance of “family” in social organizations. Families bear important economic transactions, especially the function of risk transfer. Future generations are personalized financial products such as “insurance,” “investment,” and “pension.” In areas with more Confucius temples and monasteries, the influence of traditional culture on public ideology is more obvious. In the face of health risks, even haze pollution increases the public’s risk perception level. However, influenced by traditional culture, Chinese families are not sensitive to the need for insurance, and they are more likely to cope with the possible medical burden by increasing savings and family intergenerational payments (Jin et al., 2017). Conversely, where there are fewer Confucian temples and monasteries, Chinese families will resort to more market-based measures to avoid risk, such as increasing the likelihood of buying commercial insurance. Not only that, Confucianism also influences corporate governance (Wei and Yang, 2007).

### The impact of haze pollution on demand for commercial health insurance: An analysis based on the regional differences

China has a vast territory, with noticeable economic, cultural, and customs differences in different regions. Table 6 shows the differences in institutional environments in other regions. In terms of formal institutions, whether legal environment or industry marketization level, the mean value of the eastern region is significantly higher than that of the central and western regions, and the mean value difference between the eastern region, central and western regions is significant at the level of 1%. In terms of informal institutions, the average number of Confucian temples and temples in the eastern region is also more than that in the central and western regions (except that there is no difference between the eastern and central regions). Based on the previous discussion, the institutional environment is closely related to the public’s risk cognition level, then the regional differences in the institutional environment are likely to be accompanied by the regional differences in the public’s risk cognition level. Therefore, we further discussed whether there would be significant regional differences in the impact of haze pollution on demand for commercial health insurance.

**Table 6:** Regional differences in the institutional environment.

Variable name		Inter group mean			*T*-test of mean difference between groups	
		(1)	(2)	(3)	(4)	(5)
		Eastern	Central	Western	Eastern vs. Central	Eastern vs. Western
Formal system	Legal environment	7.599	5.724	5.633	−1.875***	−1.966***
		(1.499)	(0.853)	(1.632)	(0.016)	(0.019)
	Marketization level	8.374	5.979	5.626	−2.394***	−2.747***
		(1.465)	(0.671)	(1.166)	(0.015)	(0.017)
Informal Institution	Confucian Temple	13.677	13.684	12.989	0.006	−0.689***
		(10.19)	(10.487)	(11.194)	(0.124)	(0.131)
	Temple	6.309	5.945	4.280	−0.363***	−2.029***
		(4.685)	(4.733)	(3.460)	(0.057)	(0.055)
	Observations	20,074	10,407	9,530	30,481	29,604

The values in brackets are *Z* values; *, **, *** It is significant at the level of 10%, 5%, and 1%, respectively.

From the empirical analysis results in Table 7, the coefficient of PM2.5 in column (1) is significantly positive at the coefficient of 1%, and the coefficient of PM2.5 in column (2) is significantly negative at the level of 1%, which shows that there are noticeable regional differences in the impact of haze pollution on residents’ demand for commercial health insurance, which is consistent with the hypothesis of this paper. Specifically, in the eastern region, haze pollution will increase the possibility for residents to buy commercial health insurance, while this impact is not evident in the central and western regions.

**Table 7:** Regional difference analysis of the impact of haze pollution on demand for commercial health insurance.

Variable name	(1)	(2)
	East	Midwest
PM2.5	0.138***	−2.508***
	(3.432)	(−4.563)
Income	0.111***	0.134***
	(6.763)	(7.025)
Asset	0.100***	0.137***
	(8.308)	(8.009)
Health	0.070***	0.040***
	(5.899)	(2.813)
Age	0.022***	0.044***
	(2.872)	(4.008)
Age2	−0.000***	−0.001***
	(−5.156)	(−5.425)
Gender	0.054	−0.051
	(1.384)	(−0.931)
Marry	0.058***	0.037*
	(3.410)	(1.852)
Education	0.034***	0.022***
	(6.616)	(3.584)
GDP	0.131***	0.911***
	(5.311)	(4.773)
Rural	−0.133***	−0.039
	(−2.689)	(−0.745)
_cons	−6.434***	−13.844***
	(−17.048)	(−7.217)
*N*	19,224	18,437
Pseudo *R*^2^	0.712	0.212
Wald test	15.33	26.89
Value of *p*	0.00	0.000

The values in brackets are *Z* values; *, **, *** It is significant at the level of 10%, 5%, and 1%, respectively.

In terms of the formal system, through the analysis results in Table 3, we know that if the level of the regional legal environment and market environment is high, haze pollution will increase the possibility of residents buying commercial health insurance. On the contrary, it will reduce this possibility. Combined with the analysis results of Table 6, the eastern part of the legal system level and the degree of marketization is significantly higher than in the Midwest. This explains the impact of haze pollution on the demand for commercial health insurance is stronger than in the Midwest.

In terms of the informal system, haze pollution will promote residents’ demand for commercial health insurance in areas less affected by traditional culture. On the contrary, this promoting effect is not apparent. However, the influence of traditional culture in China and the west is weaker than in the East. This is inconsistent with the results in Table 7. The informal system is not the reason for this regional difference.

In terms of control variables, we need to pay attention to the impact of regional economic development on the demand for commercial health insurance. The coefficients of (1) and (2) GDP are significantly positive at the level of 1%, which shows that regional economic development has improved the residents’ demand for commercial health insurance. However, comparing the coefficients of GDP in columns (1) and (2), the coefficient of GDP in the central and western regions is significantly greater than that in the eastern region, which shows that the development gap in the commercial health insurance industry between the west and central regions and the east region will gradually narrow due to economic development.

### Further analysis

Individual risk preference differences will affect the public’s response to haze risk. For example, different individuals have different perceptions of the profit-loss ratio of risk: some people are sensitive to the benefits of risk, while others may pay more attention to loss. For example, individuals with specific personality characteristics predict risk scenarios more positively or negatively, leading to different behavioral response styles. The existing literature found that risk attitude affects family commercial insurance participation rate (Sun and Huang, 2010). Therefore, we further analyzed the impact of haze pollution on residents’ purchase of commercial health insurance under individual risk preference. This paper uses the respondents’ answers to lottery choice and investment choice in the questionnaire to measure personal risk preference.^5^^,^^6^

In Table 8, the coefficient of PM2.5 in columns (1) and (3) is significantly positive at the level of 5% and 10%, respectively, and the coefficient of PM2.5 in columns (2) and (4) is not significant, indicating that the impact of haze pollution on demand for commercial health insurance will be different due to personal risk preference. Specifically, if individuals are risk-averse, haze pollution will promote the need for commercial health insurance. On the contrary, when individuals are risk preference, the impact of haze pollution on demand for commercial health insurance is not significant, consistent with the conclusions of the existing literature. The more risk-averse individuals are, the more willing they are to avoid risks by purchasing insurance.

**Table 8:** The impact of haze pollution on demand for commercial health insurance: an analysis based on individual risk preference.

Variable name	(1)	(2)	(3)	(4)
	Risk aversion	Risk preference	Risk aversion	Risk preference
PM2.5	0.345**	−0.248	0.323*	−0.151
	(2.06)	(−0.89)	(1.74)	(−0.50)
Income	0.092***	0.169***	0.090***	0.095*
	(3.96)	(3.41)	(3.59)	(1.85)
Asset	0.101***	0.055	0.091***	0.065
	(5.57)	(1.41)	(4.68)	(1.49)
Health	0.088***	−0.047	0.085***	−0.017
	(4.80)	(−1.02)	(4.33)	(−0.33)
Age	0.023**	0.049*	0.021*	0.026
	(2.11)	(1.93)	(1.74)	(0.95)
Age2	−0.000***	−0.001**	−0.000***	−0.000
	(−3.80)	(−2.25)	(−3.36)	(−1.40)
Gender	0.058	0.058	0.035	0.039
	(1.04)	(0.49)	(0.57)	(0.29)
Marry	0.076***	0.044	0.084***	0.119*
	(3.07)	(0.76)	(3.16)	(1.95)
Education	0.024***	0.013	0.018**	0.035*
	(3.24)	(0.89)	(2.25)	(1.96)
GDP	0.117***	0.099	0.140***	0.072
	(3.15)	(1.27)	(3.45)	(0.84)
Rural	−0.134*	−0.339	−0.067	−0.199
	(−1.70)	(−1.40)	(−0.82)	(−0.80)
region	control	control	control	control
_cons	−6.226***	−5.909***	−6.215***	−4.962***
	(−11.06)	(−5.42)	(−10.12)	(−4.18)
*N*	8,803	1,337	8,069	1,146
Pseudo *R*^2^	0.325	0.310	0.311	0.322
Wald test	4.73	0.42	4.00	0.09
Value of *p*	0.030	0.516	0.046	0.760

The values in brackets are *Z* values; *, **, *** It is significant at the level of 10%, 5%, and 1%, respectively.

In addition to purchasing commercial health insurance to deal with the risk of haze pollution, will residents take other countermeasures? The fourth part of the questionnaire asked the respondents about their family health care expenditure last year. Compared with the preventive behavior of buying insurance products, health care expenses can be regarded as an immediate response to health risks. Combined with the public’s individual risk preference, this paper further discusses the impact of haze pollution on residents’ health care expenditure.^7^

It is shown from the analysis results in Table 9 that the coefficient of PM2.5 in columns (1) and (3) is significantly positive at the level of 1%, while the coefficient of PM2.5 in columns (2) and (4) is not significant, indicating that the impact of haze pollution on residents’ health care expenditure will be significantly heterogeneous due to the difference of individual risk preference. Specifically, the risk of haze pollution will increase the health care expenditure of risk-averse individuals, but the impact on risk-averse residents is not obvious. This conclusion echoes the conclusions in Table 8, which shows that the role of individual risk preference in haze pollution events will not be different due to the timeliness of response behavior.

**Table 9:** Effect of haze pollution on family health care behavior.

**Variable name**	**(1)**	**(2)**	**(3)**	**(4)**
	**Risk aversion**	**Risk preference**	**Risk aversion**	**Risk preference**
PM2.5	0.361***	0.198	0.389***	0.259
	(2.687)	(0.864)	(2.879)	(1.096)
Income	0.116***	0.136***	0.141***	0.094***
	(6.461)	(3.449)	(7.118)	(3.267)
Asset	0.096***	0.130***	0.100***	0.125***
	(7.339)	(4.086)	(7.176)	(5.221)
Health	−0.074***	−0.065*	−0.078***	−0.058**
	(−4.559)	(−1.751)	(−4.578)	(−2.003)
Age	−0.038***	−0.014	−0.036***	−0.024**
	(−5.414)	(−0.927)	(−4.790)	(−2.039)
Age2	0.000***	0.000	0.000***	0.000*
	(5.520)	(0.800)	(4.716)	(1.721)
Gender	0.082*	0.175*	0.074	0.041
	(1.861)	(1.770)	(1.623)	(0.492)
Marry	0.033*	−0.028	0.045**	0.008
	(1.917)	(−0.565)	(2.412)	(0.236)
Education	0.060***	0.057***	0.062***	0.048***
	(10.193)	(4.326)	(9.911)	(4.476)
GDP	−0.007	−0.117*	0.029	−0.102**
	(−0.239)	(−1.895)	(0.913)	(−2.063)
Rural	−0.174**	−0.064	−0.134*	−0.439***
	(−2.491)	(−0.355)	(−1.863)	(−3.044)
Area	Yes	Yes	Yes	Yes
_cons	−3.628***	−3.270***	−4.407***	−2.576***
	(−8.445)	(−3.876)	(−9.630)	(−3.787)
*N*	8,803	1,337	8,038	2,461
Pseudo *R*^2^	0.325	0.310	0.311	0.322
Wald test	7.08	0.91	6.46	2.78
Value of *p*	0.008	0.341	0.011	0.096

The values in brackets are *Z* values; *, **, *** It is significant at the level of 10%, 5%, and 1%, respectively.

### Robustness test

To ensure the reliability of the conclusion, the robustness test is also carried out in the following aspects^8^:

Sample selection. Affected by budget constraints, families’ choices of different commercial insurance are mutually exclusive. Specifically, if families buy life insurance, it is likely to reduce the probability of purchasing health insurance. From the sample of this paper, only about 10% of the families who buy personal and commercial insurance can buy two kinds of commercial insurance simultaneously, and about 57% and 33% of the families only purchase life insurance and health insurance. This shows that most families can only buy one kind of insurance. There is an apparent mutual exclusion. To reduce the impact of this mutual exclusion on the conclusion of this paper, we only retain the samples that purchased commercial health insurance and retest the previous finding, and the results have not changed substantially.

Tool variables. Considering that many natural factors will affect haze formation, we use the regional average annual temperature and wind speed as the instrumental variables of PM2.5 and retest the conclusions of this paper, and the results have not changed substantially.

Replace the explanatory variable. Firstly, the mean value of PM2.5 in each province in the past 5 years is selected to replace the explanatory variable; Secondly, this paper uses the market-oriented total index provided by Fan Gang to replace the institutional environment index and tests the conclusions above, the results are not substantially changed.

## Conclusion

This paper finds that the level of health risk perception is an essential factor influencing commercial health insurance. The higher the level of risk perception, the higher the demand for insurance. At the same time, this result changes due to the difference in institutional environment; in a high-level legal environment and market environment, haze pollution will promote the development of commercial health insurance, while traditional culture will hinder the risk-averse function of commercial health insurance against haze pollution. Further analysis finds that the preventive measures taken by residents in the face of haze pollution will differ according to individual risk preferences. For risk-averse residents, haze pollution increases their probability of purchasing commercial health insurance. Conversely, it decreases the such probability.

Currently, the public is more concerned about the effects of haze pollution on physical health, and there seems to be no answer as to what countermeasures should be taken. The findings of this paper provide a reference for the public to use commercial health insurance to prevent haze pollution. For insurance companies, they should further improve their insurance services to meet the public’s demand for haze pollution, which will help the healthy development of the insurance industry.

## Data availability statement

The original contributions presented in the study are included in the article/supplementary material, further inquiries can be directed to the corresponding author.

## Ethics statement

Ethical review and approval was not required for the study on human participants in accordance with the local legislation and institutional requirements. Written informed consent from the patients/participants or patients/participants legal guardian/next of kin was not required to participate in this study in accordance with the national legislation and the institutional requirements.

## Author contributions

Data collection and analysis were performed by PJ. The first draft of the manuscript was written by PJ and JY. PJ and JY commented on previous versions of the manuscript. All authors contributed to the article and approved the submitted version.

## Conflict of interest

The authors declare that the research was conducted in the absence of any commercial or financial relationships that could be construed as a potential conflict of interest.

## Publisher’s note

All claims expressed in this article are solely those of the authors and do not necessarily represent those of their affiliated organizations, or those of the publisher, the editors and the reviewers. Any product that may be evaluated in this article, or claim that may be made by its manufacturer, is not guaranteed or endorsed by the publisher.

## Footnotes

^1^Baidu index is based on the search volume of Internet users in Baidu as the data, taking keywords as the statistical object, scien-tific analysis and calculation of the weighted sum of search frequency of each keyword in Baidu web search. According to the use of Baidu search sources, the search index is divided into PC search index and mobile search index.

^2^Urban and rural areas: 1 for rural areas and 0 for cities.

^3^The highest education options completed in the questionnaire are: illiterate or semi-illiterate, primary school, junior middle school, high school/technical secondary school/technical school/vocational high school, junior college, undergraduate, master, and doctor. We convert them into years of education, which are 0, 6, 9, 12, 15, 16, 19, and 22 in turn.

^4^The question to judge the health status in the questionnaire is: how is your current health status compared with your peers? 1. Very good;2. Very good;3. Good;4. General;5. No. If 1 or 2 is selected, it is regarded as good health. The virtual variable is 1; otherwise, it is 0.

^5^If there are two lottery tickets for you to choose from if you choose the first one, you have a 100% chance to get 4,000 yuan, if you choose the second one, you have a 50% chance to get 10,000 yuan, and 50% chance to get nothing, which one would you like to choose? 1. First sheet 2. Second sheet. If the respondent chooses 1, the respondent is risk-averse; otherwise, it is risk preference.

^6^If you have an asset, what kind of investment project would you like to choose? 1. Projects with high risk and high return; 2. Pro-jects with slightly higher risk and return;3. Projects with average risk and average return; 4. Projects with somewhat lower risk and return; 5. Unwilling to take any risk. If the respondent chooses 1 or 2, the risk preference is 1; otherwise, it is 0; If the respond-ent chooses 1 or 2, the respondent belongs to risk preference type; otherwise, it is risk aversion.

^7^The health care expenditure here includes the spending on health care and fitness exercise, including purchasing health care products, receiving health care physiotherapy services, running fitness cards, purchasing fitness equipment and food, asking for private education, etc. If the respondent‘s family had health care expenditure (train) last year, the value of train is 1; other-wise, the value of train is 0.

^8^Limited to space, the empirical analysis results of the robustness test are omitted here.
